# Case Report: A Case of Cotton-Wool Spots After Intravitreal Injection of Conbercept in an Infant With Incontinentia Pigmenti

**DOI:** 10.3389/fmed.2021.761398

**Published:** 2021-12-21

**Authors:** Licong Liang, Yiliu Yang, Shaochong Bu, Fang Lu

**Affiliations:** Department of Ophthalmology, West China Hospital of Sichuan University, Chengdu, China

**Keywords:** incontinentia pigmenti, conbercept, cotton-wool spots, VEGF, case report

## Abstract

**Introduction:** Incontinentia pigmenti (IP) is a rare X-linked neuroectodermal dysplasia affecting multiple organs. One of its most significant ophthalmic manifestations is retinal neovascularization due to retinal ischemia, which has been traditionally treated with laser photocoagulation or cryotherapy. The application of anti-vascular endothelial growth factor (VEGF) has been reported for the treatment of retinopathy of IP with beneficial results. However, clinicians should be aware of the possible ocular and systemic side effects of the intravitreal injection of anti-VEGF agents.

**Case Report:** A 4-month-old female infant with IP was treated with intravitreal injection of conbercept in both eyes. However, cotton-wool spots were noticed in the left eye 1 week after the injection. Laser photocoagulation was performed as an adjunct treatment. The cotton-wool spots were absorbed 1 month after the first intravitreal injection and have eventually disappeared.

**Discussion:** The cotton-wool spots, after intravitreal injection of conbercept for the treatment of IP, indicated severe retinal ischemia resulting from the neutralization of excessive VEGF, which was shown on fundus photograph and fluorescent angiography. Anti-VEGF agents could cause retinal arteriolar vasoconstriction and artery occlusion on rare occasions. The administration of anti-VEGF agents in pediatric cases with severe neovascularization and retinal ischemia should be carefully considered.

## Introduction

Incontinentia pigmenti (IP) is a rare X-linked dominant disorder that causes variable abnormalities of the skin, hair, nails, teeth, eyes, and central nervous system. It is usually prenatally lethal in males. A mutation in the affected NEMO/IKBKG (Nuclear Factor κB, Essential Modulator/inhibitor of kB kinase gamma) gene on Xq28 has been found in most cases (1).

Ophthalmic manifestations may be divided into retinal and non-retinal findings. These non-retinal associations include strabismus, conjunctival pigmentation, cataracts, ophthalmoplegia of the sixth cranial nerve, unilateral microphthalmia, and optic atrophy (2, 3). Among the abovementioned associations, retinal lesions are the most characteristic, including retinal ischemia, proliferation of new vessels with subsequent bleeding, exudation, and tractional detachment of a dysplastic retina, which resembles the retinopathy of prematurity (ROP) (4). Treatment with laser photocoagulation and cryotherapy for the proliferative vitreoretinopathy of IP has been met with variable success (5–8).

Anti-vascular endothelial growth factor (VEGF) agents have been used to treat pediatric vitreoretinal diseases, such as ROP, Coats' disease, and familial exudative vitreoretinopathy (9). Some benefits have been gained in some cases of retinopathy of IP that were treated with anti-VEGF agents (9–14). However, few adverse effects have been reported. Thereafter, we will report a case of the cotton-wool spots, following intravitreal anti-VEGF therapy in a patient with retinopathy of IP.

## Case Report

**Figure 1:**
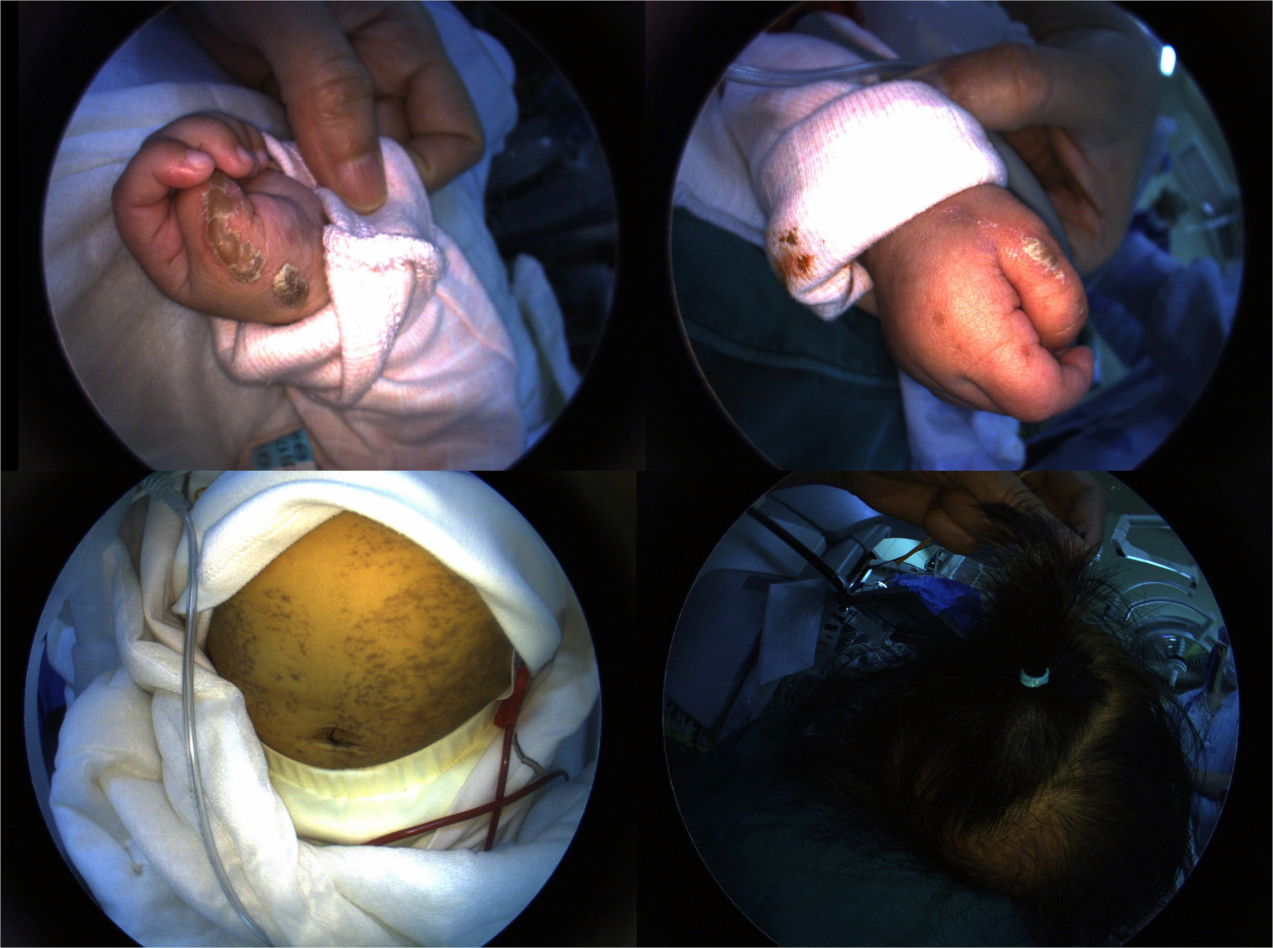
Typical neonatal rash on the infant's skin.

**Figure 2:**
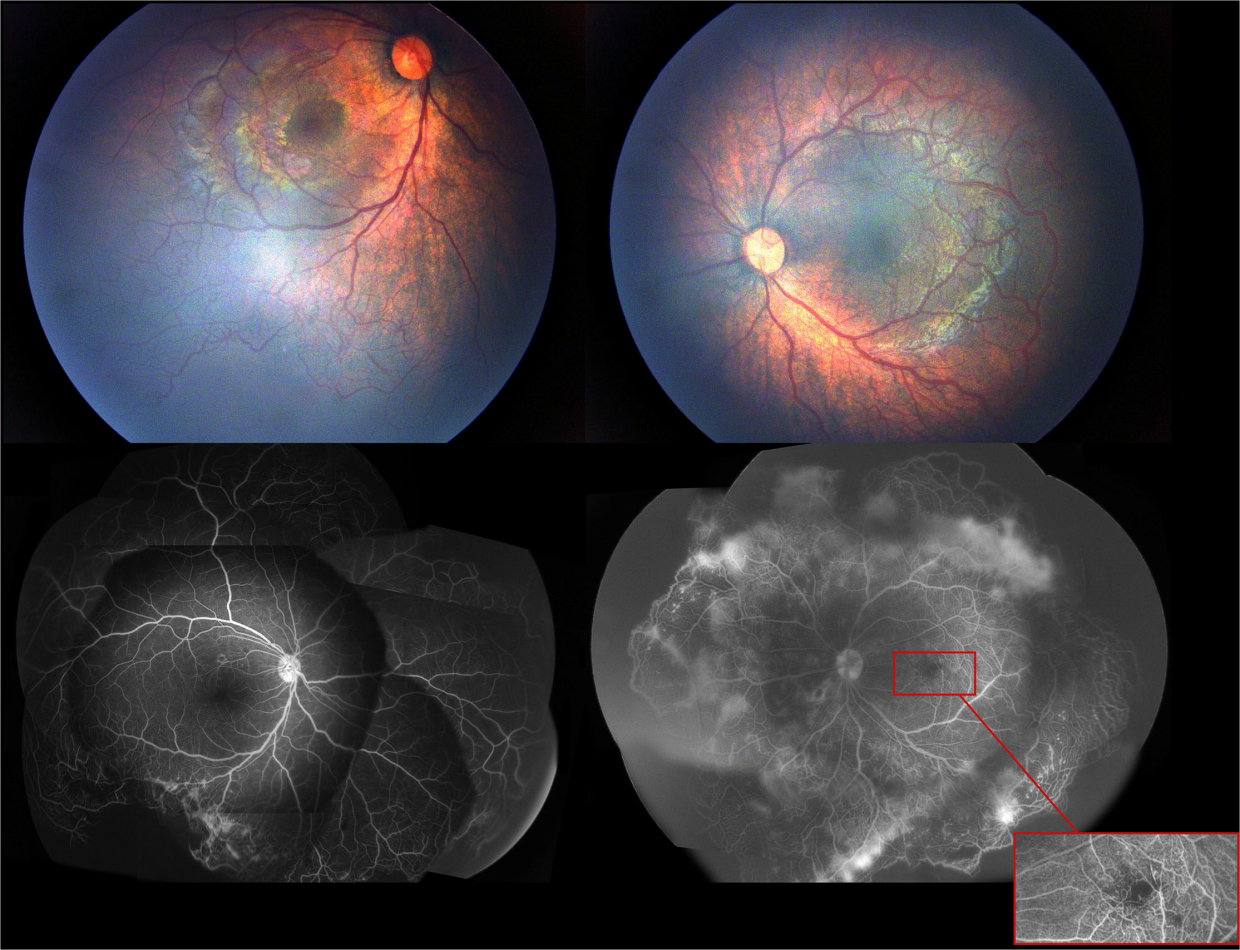
The first exam of fundus photography and fluorescein angiography image showed incomplete peripheral vascularization, retina neovascularization, foveal hypoplasia, and fluorecsein leakage.

A 4-month-old female infant with a birth weight of 2,400 g and a gestational age (GA) of 37^+1^ weeks was diagnosed with IP by typical neonatal rash and truncal hyperpigmentation (Figure 1). However, her twin sister was healthy though both of them were fertilized *in vitro*. Fundus photography and fluorescein angiography (FA), using a RetCam III wide-angle fundus imaging system (Clarity Medicla Systems, Pleasanton, CA, USA), showed incomplete peripheral vascularization, retina neovascularization, foveal hypoplasia, and fluorescein leakage in both eyes, which were more pronounced in the left eye (Figure 2). Intravitreal injection with 0.25 mg conbercept (Lumitin® Chengdu Kanghong Biotech Co, Ltd, Chengdu, China) was administered to each eye after obtaining informed consent from her parents. The patient was discharged and scheduled to return to the outpatient clinic in a week.

**Figure 3:**
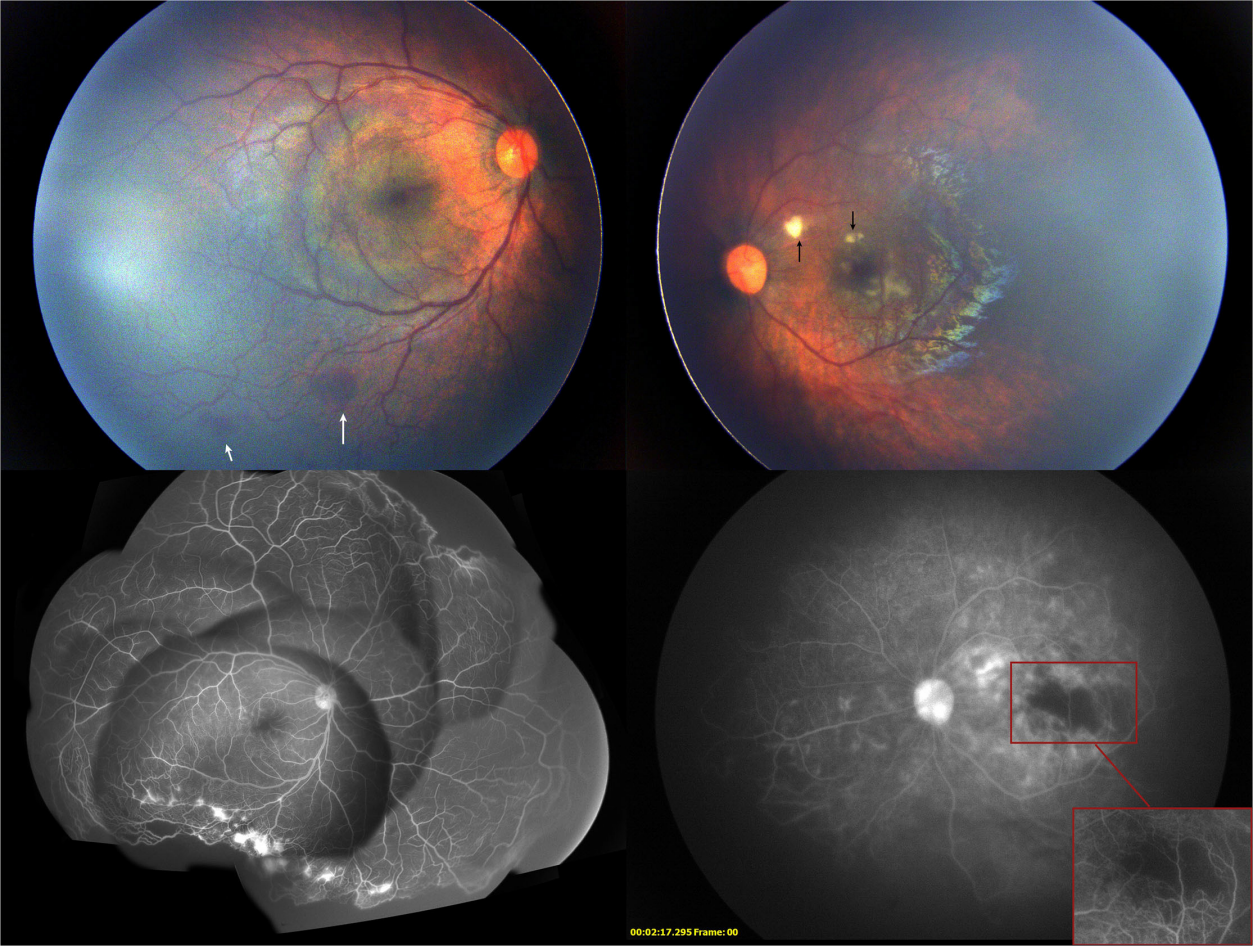
Two weeks after the first intravitreal injection of conbercept, the fundus photography showed mild hemorrhage (white arrow) on the peripheral retina in the right eye and cotton-wool spots (black arrow) near the macular in the left eye. Fluorescein angiography showed the enlargement of the non-perfused region of the retina in the left eye and the regression of neovascularization in both eyes.

One week after the injection, the cotton-wool spots, which were one disc diameter away from the fovea in the superior papillomacular bundle and vascular constriction in the posterior pole, were noticed in the left eye. Further examination revealed mild hemorrhage in the inferior peripheral retina of the right eye. FA showed complete regression of neovascularization with severe vascular constriction, circumferential peripheral retinal non-perfusion, macular ischemia, and diffuse leakage of the remaining retinal vessel in the late phase in the left eye. The peripheral non-perfusion and neovascularization in the right eye remained unchanged (Figure 3). At this point, 532 nm laser photocoagulation with indirect ophthalmoscope delivery system was performed on the peripheral non-perfusion and avascular retina in the left eye. The laser settings used were a power of 120–160 mW and duration of 200–400 ms and the number of spots was 497. The ischemic retina was not fully covered due to the development of corneal edema during the procedure. The patient was followed closely every week afterward.

**Figure 4:**
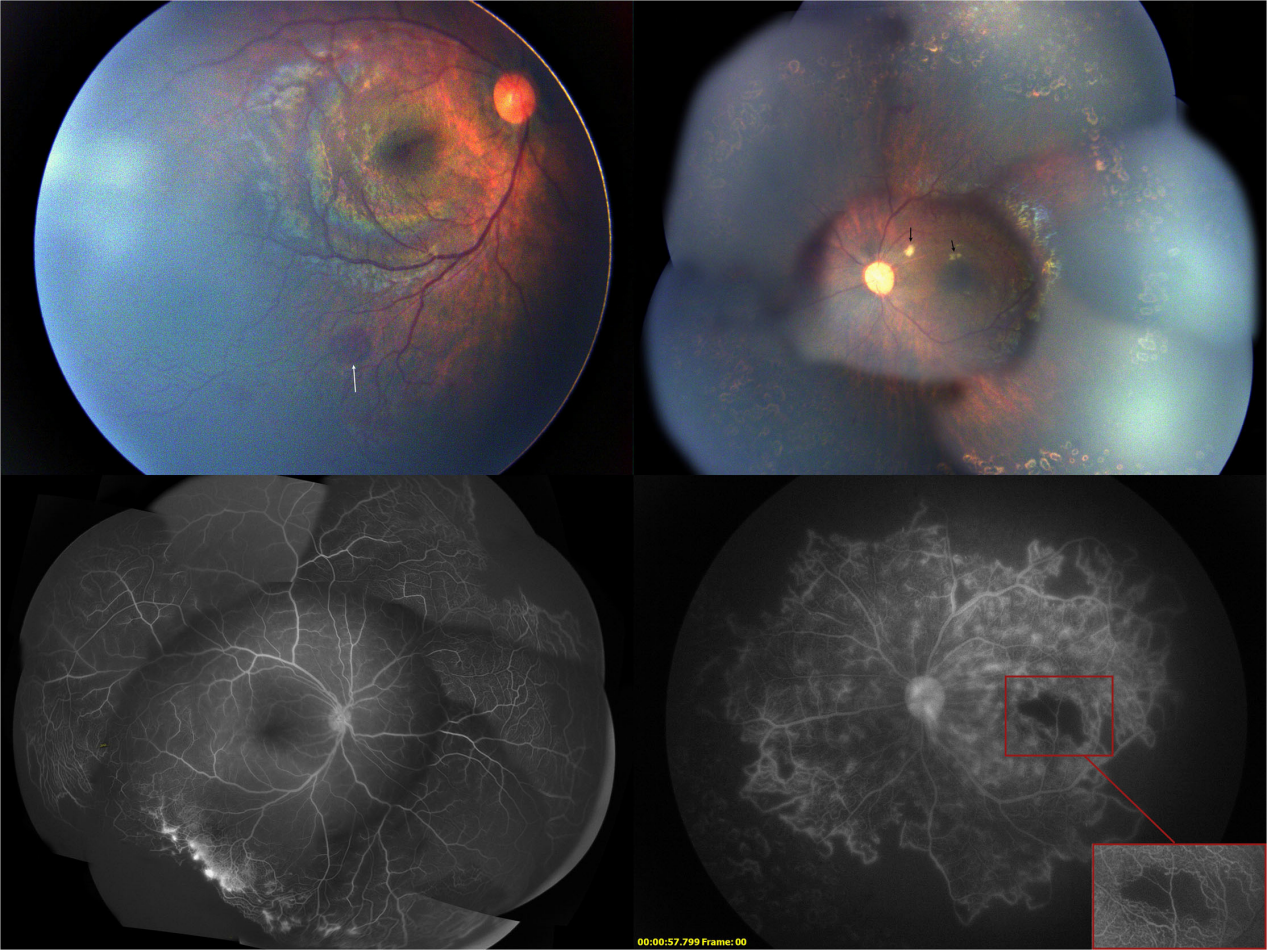
One month after the first intravitreal injection of conbercept, the fundus photograph showed the absorption of the cotton-wool spots and the fluorescein angiography showed regression of neovascularization.

One month after the initial injection, cotton-wool spots in the left eye decreased, and the vascular constriction gradually recovered. The ischemia of the macula and retina in both eyes remained unchanged. Another session of 532 nm laser photocoagulation with 238 spots to the inferior ischemic retina near the posterior pole was performed on the left eye (Figure 4). The second intravitreal injection with 0.25 mg conbercept was administered to the right eye for the recurrence of retinal neovascularization.

**Figure 5:**
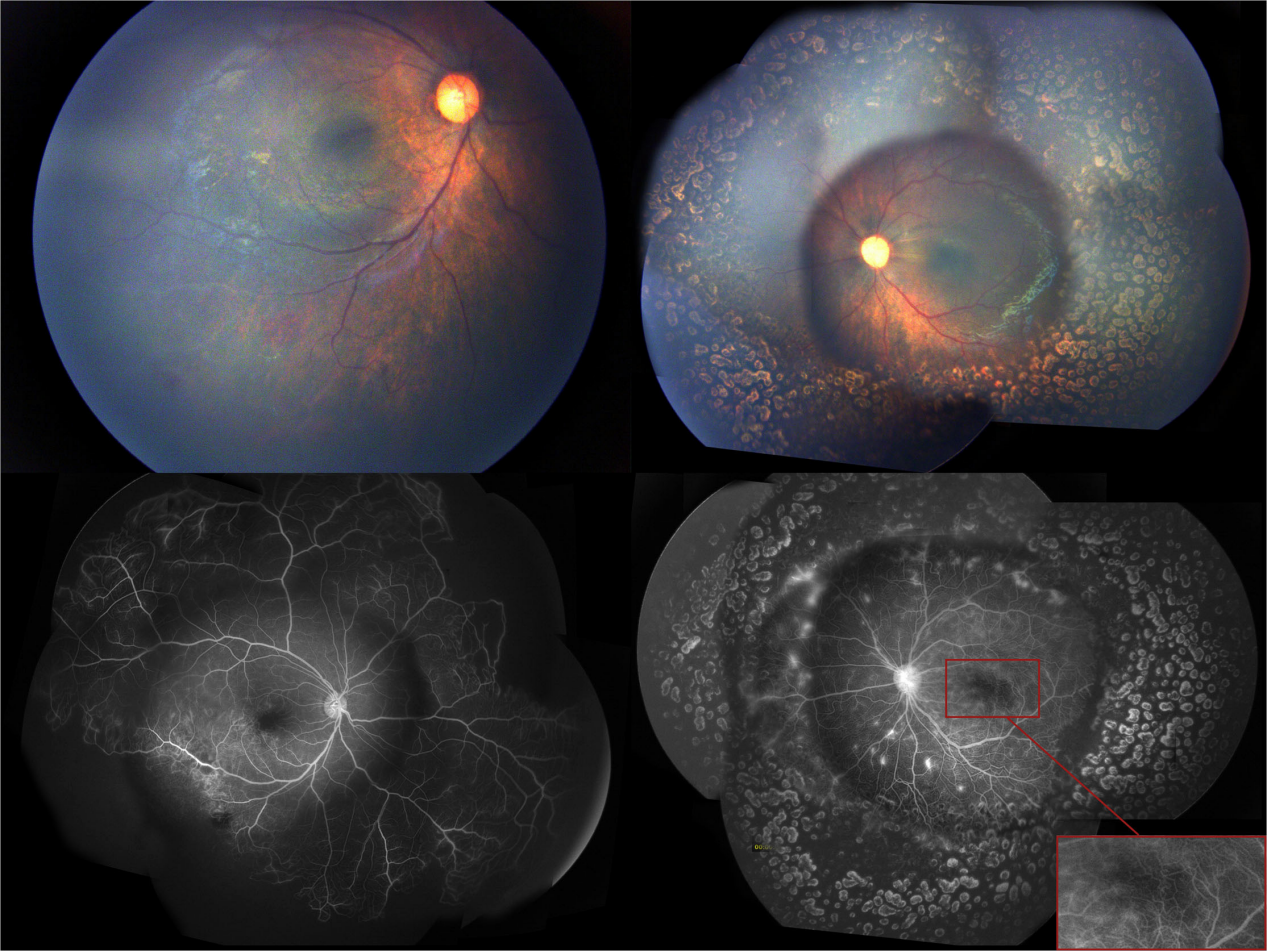
Two months after the first intravitreal injection of conbercept, the fundus examination showed the disappearance of the cotton-wool spots in the left eye, and the fluorescein angiography showed further regression of neovascularization in the right eye.

Two months after the first treatment, fundus examination of the left eye showed total absorption of cotton-wool spots. FA showed that macular ischemia was improving, and peripheral retinal perfusion has recovered with neovascularization recurrence. Additional 532 nm laser treatment was applied to the remaining inferior temporal avascular retina of the left eye (Figure 5).

**Figure 6:**
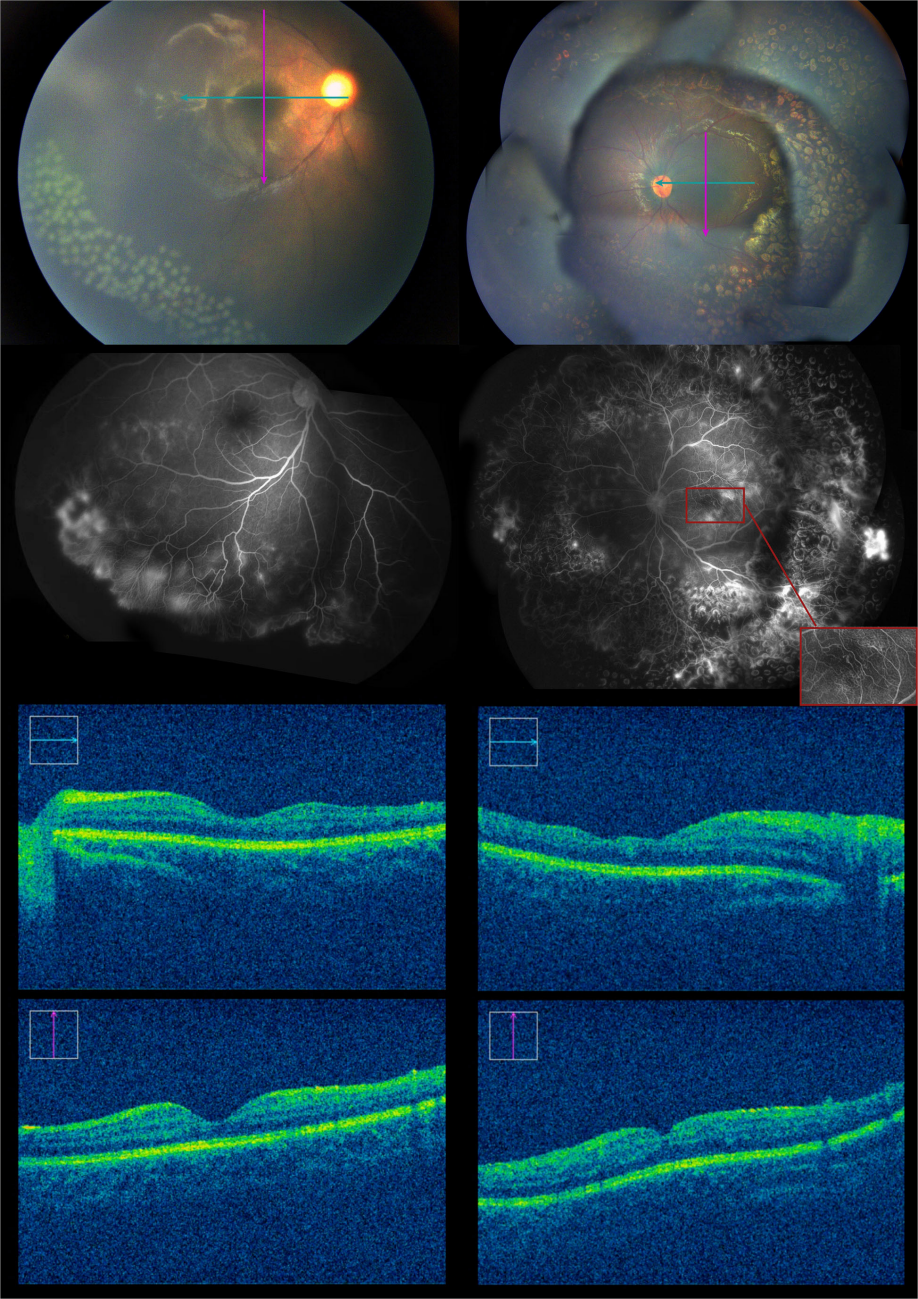
Eight months after the first intravitreal injection of conbercept, the fundus examination showed some blood vessels grew into the laser spot region of the retina, and the fluorescein angiography showed some activation of neovascularization in the left eye. There was no significant change in the right eye. About 100 laser photocoagulation spots then could be seen in the avascular area of the right eye.

Eight months after the initial anti-VEGF treatment. In the left eye, the vessels at the border of the peripheral avascular area grew into the lasered retina, with some neovascular membranes showing hyperfluorescein. However, the new vessels presented on the last FA regressed. In the right eye, FA showed extensive leakage of the vessels in the inferotemporal quadrant. The fourth session of 532 nm laser treatment was administrated on the neovascularization and on the leaking areas in the left and right eyes, respectively. We also performed OCT (CALLISTO eye, Carl Zeiss Meditec AG, Oberkochen, Germany) with a microscope. The OCT showed that the fovea of the left eye was thinner than that of the right eye, especially the temporal macula (Figure 6).

We recommend a long-term follow-up every 3 months in the clinic moving forward. Laser photocoagulation or surgery should be considered if neovascularization recurs, or if retinal traction develops. Presently, the condition remains stable with no progression of neovascularization or non-perfusion. Further anti-VEGF therapy may not be the best choice for the patient because of the short effective period. In subsequent treatment, laser photocoagulation rather than anti-VEGF drugs is recommended.

## Discussion

Pediatric anti-VEGF therapy has gradually extended from being mainly the treatment of retinopathy of prematurity to a broader spectrum of retinal diseases when avascular retina and neovascularization occur. Over the last decade, a few case reports have shown beneficial outcomes in the treatment of retinopathy of IP using anti-VEGF agents when the lesions are refractory to traditional laser and cryotherapy (10). Since then, anti-VEGF treatment for retinopathy of IP has been gaining popularity with increasing experience in clinical usage in pediatric cases (9–14). However, fine-tuning of the treatment protocol and dosage titration have not yet been developed.

The current case report indicated that the response of the same patient with different extents of the disease could respond with large variation using the same dosage of anti-VEGF intravitreal injection. The limited peripheral avascular area and neovascularization in the right eye showed a favorable response to anti-VEGF treatment. With additional laser photocoagulation, the lesion remained stable. However, retinal ischemia deteriorated in the left eye after anti-VEGF injection, as was indicated by the development of cotton-wool spots in the vicinity of the macula and attenuation of the retinal vasculature in the midperiphery and macular region. These findings in the left eye suggested that the vascular contraction and regression induced by anti-VEGF agents affected not only the abnormal neovasculature, but also the normal retinal circulation, which resulted in a severe retinal ischemic state.

With laser photocoagulation and the gradual attenuation of the effect of conbercept, vascular constriction and the resulting retinal ischemia gradually recovered during the close follow-up. This case reminded us that anti-VEGF agents are mainly targeted to attenuate the abnormal vessels but not the underlying retinal ischemia. Therefore, in the presence of extensive retinal ischemia, the administration of intravitreal anti-VEGF agents should proceed with caution.

In this case, we suspected that severe retinal neovascularization and a complete avascular retina, except the posterior pole, as initially shown in the left eye, were induced by upregulation of VEGF, which is also the pathogenesis of IP retinal vasculopathy (15). With the sudden inhibition of VEGF after intravitreal conbercept, neovascularization regressed effectively and tragically with the occlusion of physiological retinal vascular circulation. Then, cotton-wool spots and retinal vascular attenuation were inspected 1 week after anti-VEGF injection. As conbercept was gradually metabolized, VEGF in the left eye returned, and cotton-wool spots were gradually absorbed in the following 7 weeks. Vascular attenuation was also relieved.

The overproduction of VEGF in ischemic retinal cells and other angiogenic factors is one of the major causes of the formation of abnormal new vessels and fibrotic tissue. The rationale of the application of anti-VEGF therapy in pediatric retinal diseases is to attenuate abnormal neovascularization while allowing the normal vasculature to develop (16). The adverse effects of anti-VEGF on normal retinal circulation should be considered in cases with severe retinal ischemia. Previous reports showed that anti-VEGF agents could cause retinal arteriolar vasoconstriction and artery occlusion on rare occasions (17–19). Aflibercept, an Fc fusion protein, has been reported to cause retinal arterial occlusion and retinal vasculitis (20). Data from the post-marketing aflibercept Global Safety Database showed that the rate of retinal arterial occlusion, vasculitis, or severe vision loss was 0.9 per 10,000 aflibercept injections (21). A case of complete arrest of retinal vascular growth until 7 months after receiving intravitreal injection of aflibercept for aggressive posterior ROP might indicate that aflibercept can affect normal vascular growth (22). However, there were no FA images in this publication. A retrospective study estimated that 22.8% of eyes with central retinal vein occlusion (CRVO) developed cotton-wool spots after an initial aflibercept injection and indicated that cotton-wool spots were reversible once the circulatory disturbance of the capillary bed was resolved (23).

Conbercept is also an Fc-fusion protein. The common ocular adverse events of conbercept are transient spikes of intraocular pressure, vitreous floaters, cataracts, conjunctival hemorrhage, and corneal inflammation with no permanent consequences (24). The occurrence of cotton-wool spots or retinal arterial occlusion that is identified after the intravitreal injection of conbercept has not been reported until now. Although adverse ocular events are rare after intravitreal conbercept in IP patients, frequent assessment of the treatment should be emphasized.

In our case, the cotton-wool spots diminished 2 months after the first injection. We speculated that the disappearance of cotton-wool spots indicated reperfusion of the retinal circulation. Immediate laser photocoagulation could be an effective option to prevent further damage caused by retinal ischemia. In pediatric retinal vascular diseases with severe neovascularization and ischemia, we should not only be alert for fibrovascular tractional retinal detachment after intravitreal injection of anti-VEGF agents, but also pay attention to the aggravation of retinal ischemia after the treatment (25). Cotton-wool spots could be an indicator of the aggravation of retinal ischemia. The administration of anti-VEGF in pediatric cases with severe neovascularization and retinal ischemia should be carefully considered and closely followed if applied.

## Data Availability Statement

The original contributions presented in the study are included in the article/supplementary material, further inquiries can be directed to the corresponding author.

## Ethics Statement

Written informed consent was obtained from the minor(s)' legal guardian/next of kin for the publication of any potentially identifiable images or data included in this article.

## Author Contributions

LL collected clinical information, consulted literature, and then wrote this case report. YY collected the latest information of this case. SB edited this case. FL provided this case and offered guidance. All authors contributed to the article and approved the submitted version.

## Funding

Funding provided by Beijing Bethune Charitable Foundation (HX-H1703040).

## Conflict of Interest

The authors declare that the research was conducted in the absence of any commercial or financial relationships that could be construed as a potential conflict of interest.

## Publisher's Note

All claims expressed in this article are solely those of the authors and do not necessarily represent those of their affiliated organizations, or those of the publisher, the editors and the reviewers. Any product that may be evaluated in this article, or claim that may be made by its manufacturer, is not guaranteed or endorsed by the publisher.
